# Pilot study examining anti-factor Xa levels for heparin monitoring and outcomes in patients with cerebral venous thrombosis

**DOI:** 10.3389/fmed.2024.1317246

**Published:** 2024-01-26

**Authors:** Yasaman Pirahanchi, Kristin Salottolo, Christian Burrell, Xu Tang, David Bar-Or, Russell Bartt

**Affiliations:** ^1^Neurology Department, Swedish Medical Center, Englewood, CO, United States; ^2^Trauma Research Department, Swedish Medical Center, Englewood, CO, United States

**Keywords:** cerebral venous thrombosis, unfractionated heparin, anti-factor-Xa, monitoring, outcomes

## Abstract

**Objective:**

There are no studies to date that examine the association between anti-factor-Xa (AFXa)-based heparin monitoring and clinical outcomes in the setting of cerebral venous thrombosis (CVT).

**Methods:**

This pilot study included adults aged ≥18 admitted with CVT between 1 January 2018 and 1 January 2021, who were treated with unfractionated heparin (UFH) and were monitored via AFXa-based nomogram within 24 h of arrival. Comparisons were made between patients with AFXa levels within the target therapeutic range (0.25–0.5 IU/mL) and patients whose levels were not within the therapeutic range within 24 h of arrival; the time (hours) from arrival to reach the therapeutic range was also examined. Outcomes were length of stay (LOS) in the hospital, major (actionable) bleeding events, and discharge home (vs. higher acuity location). Continuous data are reported in the form of the median (interquartile range).

**Results:**

Among 45 patients, treatment with UFH was initiated 2 (1–11) h after arrival, and the majority (84%) of UFH infusions did not need dose adjustment. AFXa assays were conducted every 6 (5.5–7) h. Thirty patients (67%) fell within the therapeutic range. Outcomes were similar for patients with levels within the therapeutic range vs. not: major bleeding events, 10% vs. 0% (*p* = 0.54); discharge home, 77% vs. 80% (*p* = 1.0); LOS, 5 days in each group (*p* = 0.95). There was also no association between outcomes and time to reach the therapeutic range.

**Conclusion:**

Our findings demonstrate the practicability of monitoring UFH based on AFXa values in this population of patients with CVT, but reaching target AFXa levels within 24 h of arrival may not necessarily be prognostic.

## 1 Introduction

Cerebral venous thrombosis (CVT) is a rare cause of stroke that occurs in less than 1 per 100,000 people annually, and although it is generally associated with favorable outcomes, it still causes significant long-term disability and approximately 10% mortality (1, 2).

CVT is treated with systemic anticoagulant therapy regardless of the presence of pre-treatment intracerebral hemorrhage (ICH) (3). American Heart Association/ American Stroke Association guidelines recommend intravenous unfractionated heparin (UFH) or subcutaneous low-molecular-weight heparin (LMWH) (4). Heparin exerts its anticoagulant effects primarily via inactivation of thrombin and factors Xa, IXa, and XIa and XIIa, preventing fibrin formation and inhibiting thrombin-induced activation of platelets and coagulation factors (5).

LMWH demonstrates predictable pharmacodynamics and pharmacokinetics, rendering routine therapeutic drug monitoring (TDM) for anticoagulation effects unindicated in the majority of patients. However, TDM may be indicated in certain patient populations receiving LMWH to enhance the predictability of optimal dosing, including patients with hepatic or renal insufficiency, pregnant patients, octogenarians, and patients with extreme body weight (<40 kg or > 150 kg) (5, 6).

An advantage of UFH over LMWH in the context of CVT is a more immediate anticoagulant effect as well as a shorter elimination half-life (5). However, reaching an optimal therapeutic dose of UFH can be challenging due to unpredictable pharmacokinetics, interpatient variability, and anarrow therapeutic index, and TDM is indicated (7).

UFH monitoring may lead to fewer dose adjustments and less time to reach the therapeutic range, whereas suboptimal or overdosing may lead to poorer outcomes, including thromboembolic complications, major hemorrhage, and thromboembolic recurrence (8–10). While direct monitoring of serum concentration of UFH is not possible, there are several surrogate tests available for UFH monitoring, including activated partial thromboplastin time (aPTT), activated clotting time, plasma heparin concentration, and anti-factor Xa (AFXa). AFXa assays have been shown to be superior to aPTT-based protocols (10–12) and are less sensitive to reagents and less influenced by external factors that are also known etiologies of CVT, such as pregnancy and the presence of lupus anticoagulant (13, 14).

To our knowledge, there is only one published study examining AFXa monitoring in the setting of CVT, but this study was performed in children (14). There are no studies to date that have primarily focused on identifying and describing the practicability of UFH monitoring specifically in the setting of adult CVT. This pilot study describes adults with CVT treated with UFH and monitored via AFXa assay and reports on the association between AFXa monitoring and clinical outcomes.

## 2 Methods

### 2.1 Design, setting, and population

**Figure 1:**
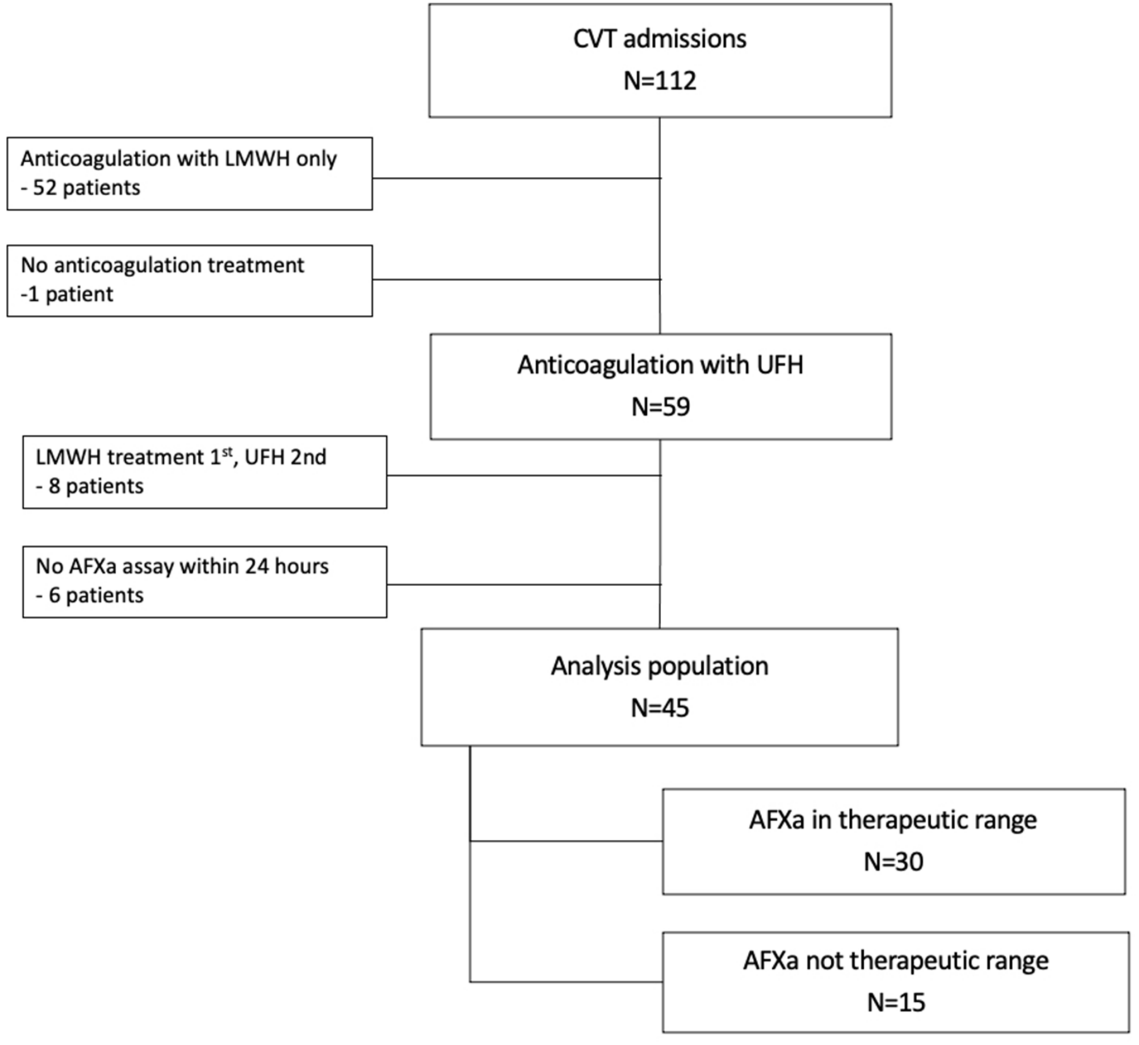
Study population. CVT, cerebral venous thrombosis; UFH, unfractionated heparin; LMWH, low molecular weight heparin; AFXa, anti-factor-Xa.

This retrospective pilot study included adults (≥18 years old) who were admitted to a comprehensive stroke center between 1 January 2018 and 1 January 2021 for CVT. AFXa assays were conducted at our institution as a surrogate marker to measure the extent of anticoagulation with UFH. Patients were excluded if they did not initially receive treatment with UFH or if they did not undergo at least one documented AFXa assay within 24 h of arrival (Figure 1). The study received IRB approval with waiver of consent. The initial study population was identified through the hospital’s neurology patient registry database. Data were extracted from electronic health records by clinicians (Y.P., X.T., and L.D).

### 2.2 Anticoagulation protocol

The institutional protocol for anticoagulation treatment of CVT is to begin either LMWH or UFH within 2 h of arrival; for the latter, low-intensity heparin infusion is used for stroke. The standard heparin concentration is 100 units/mL with an initial infusion rate of 12 units/kg/h, with weight-based calculations for specific infusion rate and for the heparin bolus dose (if ordered).

Coagulation monitoring for UFH via AFXa assay began at the end of 2017 and was in place throughout the study period. The anti-factor Xa heparin assay is a chromogenic assay that measures the factor Xa-neutralizing capacity of heparin (15). When antithrombin binds to factor Xa in a sample containing heparin, it forms a complex, which can lead to underestimation of the concentration of active factor Xa in the assay. In the anti-Xa assay, the goal is to measure the residual, unbound factor Xa activity in a patient’s blood plasma or serum. The assay uses a substrate that is cleaved by factor Xa, leading to a color change that can be quantified. The degree of color change is directly proportional to the amount of unbound factor Xa in the sample (5).

Our hospital uses the Siemens INNOVANCE anti-Xa assay, which is an automated chromogenic anti-Xa assay for quantitatively determining the activity of UFH (16). Quality control checks are performed at the beginning of every shift to ensure the test is running consistently. AFXa assays are ordered every 6 h after initiation of UFH infusion or any dosage change; aPTT assays may have also been performed, but not at standardized intervals or to assess coagulation. Adjustments to the infusion as determined in our hospital-based guidelines are shown in Table 1. The therapeutic range for UFH is 0.25–0.5 IU/mL, with the intent to achieve a therapeutic range within 18–24 h.

**Table 1:** Hospital-based protocol for heparin rate adjustment based on anti-factor Xa (AFXa) value.

AFXa value (IU/mL)	Stop infusion	Bolus	Rate change (units/kg/h)	Repeat assay in:
Less than 0.15	No	No	↑ 3 units/kg/h	6 h
0.15–0.24	No	No	↑ 2 units/kg/h	6 h
0.25–0.5	No change (Therapeutic Range)			6 h*
0.51–0.65	No	No	↓ 1 unit/kg/h	6 h
0.66–0.8	60 min	No	↓ 2 units/kg/h	6 h
0.81 or higher	120 min	No	↓ 3 units/kg/h	6 h

^*^Repeat every 24 h if two consecutive assays are in the therapeutic range. kg/h, kilograms per hour.

### 2.3 Exposure and outcomes

AFXa values were examined in two ways: (1) whether the patient reached the therapeutic range within 24 h of arrival (yes vs. no), defined as an AFXa value between 0.25 and 0.5 IU/mL; and (2) the time (hours) from arrival to the first AFXa within the therapeutic range. The cutoff point of 24 h was based on the institutional protocol and previous studies (6).

Study outcomes were examined during the hospitalization period and included major and minor bleeding events, other complications (e.g., ischemic or hemorrhagic stroke, intracerebral hemorrhage secondary to acute stroke treatment, groin puncture, or hospital complications/events), LOS in the hospital (days), discharge disposition, and in-hospital mortality. Bleeding events were defined *a priori* and extracted from records for this study by research clinicians (Y.P., X.T., and L.D). Major bleeding events were considered if there was discontinuation of the anticoagulant, a reversal agent was used, or the patient needed transfusion or surgery. Minor bleeding was defined as overt bleeding that was not actionable (the patient experienced symptoms but no action was needed, i.e., no discontinuation of anticoagulant, no use of a reversal agent, and no blood transfusion or surgery required). Discharge disposition was categorized as home/home health care; care facility (including rehabilitation and long-term care facilities); or morgue.

### 2.4 Statistical analysis

All analyses were performed using SAS (Cary, NC) version 9.4. The threshold for statistical significance was *α* < 0.20; this level of significance is appropriate for small sample sizes and pilot studies rather than the conventional *α* < 0.05 (17, 18). Continuous data are presented in the form of the median (interquartile range). There was no imputation of missing data. The associations between outcomes and achievement of AFXa within the therapeutic range within 24 h (yes vs. no) were analyzed via chi-square tests (categorical outcomes) and a Wilcoxon rank-sum test (LOS in the hospital). The associations between outcomes and time (hours) to reach the therapeutic range were analyzed via Wilcoxon rank sum tests (categorical outcomes) and the Spearman correlation coefficient (LOS).

## 3 Results

### 3.1 Patients

There were 45 patients with CVT in the analysis population (Figure 1). The median age was 42 (34–57) years, and the majority of patients were female (53%), transferred in (69%), presented with headache (76%), and had multiple veins/sinuses involved (Table 2). Outcomes were generally favorable. The majority of patients (78%) were discharged home, 20% were discharged to a care facility, and one patient died. There were three documented major bleeding events (6%); surgery, a reversal agent, and discontinuation of the UFH infusion were each required in one case. Four additional patients had a minor bleeding event. One patient developed an additional complication, a groin hematoma, following intra-arterial therapy. The median LOS in the hospital was 5 (4–8) days.

**Table 2:** Patient characteristics.

Covariate	AFXa in the therapeutic range			
	Overall (*n* = 45)	Yes 30 (67%)	No 15 (33%)	*p*-value
Age, median (IQR)	42 (34–57)	40.5 (34–54)	46 (39–65)	0.36
Male sex	21 (46.7)	15 (50.0)	6 (40.0)	0.53
White race	25 (55.6)	16 (53.3)	9 (60.0)	0.67
Sinus involvement^*^				
Superior sagittal sinus	20 (44.4)	15 (50.0)	5 (33.3)	0.29
Straight sinus	4 (8.9)	2 (6.7)	2 (13.3)	0.59
Transverse sinus	30 (66.7)	19 (63.3)	11 (73.3)	0.50
Sigmoid sinus	29 (64.4)	18 (60.0)	11 (73.3)	0.38
Jugular vein	14 (31.1)	8 (26.7)	6 (40.0)	0.50
Other	11 (24.4)	8 (26.7)	3 (20.0)	0.73
Endovascular therapy	22 (48.9)	17 (56.7)	5 (33.3)	**0.14**
Signs/symptoms^*^				
Headache	34 (75.6)	22 (73.3)	12 (80.0)	0.73
Hemorrhagic CVT	6 (13.3)	5 (16.7)	1 (6.7)	0.65
Seizure	14 (31.1)	11 (36.7)	3 (20.0)	0.32
Altered mentation	8 (17.8)	6 (20.0)	2 (13.3)	0.70
Nausea or vomiting	4 (8.9)	3 (10.0)	1 (6.7)	1.0
Motor impairment	8 (17.8)	5 (16.7)	3 (20.0)	1.0
Sensory or visual	9 (20.0)	6 (20.0)	3 (20.0)	1.0
Transferred in	31 (68.9)	21 (70.0)	10 (66.7)	1.0

IQR, interquartile range; CVT, cerebral venous thrombosis. *More than 1 allowed, will not total 100%. Bolding denotes statistical significance at *p* < 0.20.

The median time to initiate UFH was 2 (1–11) h after arrival. Approximately 24% of patients received an initial bolus, with a median of 2,000 (1,000–3,000) units. The median continuous infusion was 250 (250–250) units/kg. The majority of patients (84%) did not need UFH volume adjustment, and the majority of heparin infusions (84%) did not need volume adjustment. Most patients (84%) were transitioned to LMWH, approximately 27 (17–61) h after arrival. Fifteen patients received LMWH within 24 h of arrival.

AFXa assays were conducted every 6 (5.5–7) h; there were 4 (2–8) draws per patient. The median time from arrival to the first AFXa assay was 7.5 (3–9) h.

### 3.2 Therapeutic range within 24 hours

There were 30 patients (66.7%) who were within the therapeutic range within 24 h of arrival. The median time to reach the therapeutic range was 9 (5–15.5) h after initiation of UFH, or 14 (9–20) h after arrival. The heparin infusion volume was adjusted for three patients (10%) who were within the therapeutic range within 24 h of arrival and four patients (27%) who were not within the therapeutic range within 24 h of arrival.

Patient demographics and presenting characteristics were similar for patients who were in the therapeutic range vs. those who were not, with the exception that patients in the therapeutic range were more likely to receive endovascular therapy than those who were not (56.7% vs. 33.3%, *p* = 0.14) (Table 2).

More than half (8 of 15) of the patients who were not within the therapeutic range within 24 h did not reach the therapeutic range at any point during their hospital stay. Among the 15 patients who were not within the therapeutic range within 24 h, 8 patients were supratherapeutic (AFXA >0.81), 3 patients were subtherapeutic (AFXA <0.15), and 4 patients had AFXa values that were outside the therapeutic range but were in the range 0.15–0.49 or 0.51–0.80.

### 3.3 Association with outcomes

There was no association between study outcomes and whether the patient achieved AFXa values within the therapeutic range within 24 h or not (Table 3). Major bleeding events were reported in 10% (*n* = 3) of patients who reached the therapeutic range vs. 0% for those who did not within within 24 h of arrival (*p* = 0.54); the rate of home discharge was 77% for those who were within the therapeutic range vs. 80% who were not (*p* = 1.0); and LOS in the hospital was 5 days for each group (*p* = 0.95). Similarly, for the subset of 22 patients who underwent endovascular therapy, there was no association between study outcomes and whether the patient achieved AFXa values within the therapeutic range within 24 h or not (Table 3).

**Table 3:** Association between therapeutic anti-factor Xa (AFXa, 0.25–0.5 IU/mL) within 24 h of arrival and outcomes.

Outcome	Therapeutic AFXa: all patients (*n* = 45)			Therapeutic AFXa: endovascular therapy subset (*n* = 22)		
	Yes 30 (67%)	No 15 (33%)	*p*-value	Yes 17 (77%)	No 5 (23%)	*p*-value
Discharge disposition, *n* (%)			0.77			0.76
Home	23 (76.7)	12 (80.0)		11 (64.7)	4 (80.0)	
LTAC/Rehab	6 (20.0)	3 (20.0)		5 (29.4)	1 (20.0)	
Died	1 (3.3)	0 (0)		1 (5.9)	0 (0)	
Major bleed, *n* (%)	3 (10.0)	0 (0)	0.54	2 (11.8)	0 (0)	1.0
Minor bleed, *n* (%)	2 (6.7)	2 (13.3)	0.59	1 (5.9)	1 (20.0)	0.41
Median LOS (IQR)	5 (3–9)	5 (4–8)	0.95	6 (4–12)	5 (4–9)	0.69

*Among 15 patients not in the therapeutic range, 6 patients were supratherapeutic (AFXA > 0.50), 4 patients were subtherapeutic (AFXA < 0.25), and 5 patients had AFXa values that were both super- and subtherapeutic. LTAC, long-term acute care; IQR, interquartile range. Major bleeds were actionable, whereas minor bleeds required no action.

There were also no major differences in outcomes for patients based on the degree to which patients’ AFXa values fell within the therapeutic range within 24 h (Table 4).

**Table 4:** Association between anti-factor Xa values (IU/mL) within 24 h of arrival and outcomes.

Outcome	Therapeutic	Not in the therapeutic range			*p* value
	AFXa 0.25–0.50 *N* = 30	AFXa <0.15 *N* = 3	AFXa >0.81 *N* = 8	AFXa >0.15 and < 0.81 *N* = 4	
Discharge disposition, *n* (%)					
Home	23 (76.7)	2 (66.7)	7 (87.5)	3 (75.0)	0.98
LTAC/Rehab	6 (20.0)	1 (33.3)	1 (12.5)	1 (25.0)	
Died	1 (3.3)	0 (0.0)	0 (0)	0 (0)	0.92
Major bleed, *n* (%)	3 (10.0)	0 (0.0)	0 (0)	0 (0)	0.66
Minor bleed, *n* (%)	2 (6.7)	1 (33.3)	1 (12.5)	0 (0)	0.41
Median LOS (IQR)	5 (3–9)	8 (6–16)	5 (3.5–7)	4.5 (3–6)	0.36

LTAC, long-term acute care; IQR, interquartile range. Major bleeds were actionable, whereas minor bleeds required no action.

There was also no association between study outcomes and the amount of time taken (hours) to reach the therapeutic range. The median time to reach the therapeutic range was 11 (8–15) h for those discharged home vs. 14 (8–20) h for discharge to a care facility or morgue (*p* = 0.49), and 13 (9–20) h for patients who developed a major bleeding event vs. 12 (8–16) h for those without a major bleeding event (*p* = 0.68). There was also no correlation with LOS in the hospital (*p* = 0.77).

## 4 Discussion

In this pilot study of patients with CVT, we examined whether dose-adjusted monitoring of UFH using an AFXa assay was associated with clinical outcomes (LOS, major bleeding events, or favorable discharge disposition). Outcomes were similar for patients whose levels were within the therapeutic range and those who were not within the therapeutic range within 24 h of arrival. Taking less time to reach therapeutic AFXa values was also not associated with better outcomes, although poor outcomes were uncommon. One interpretation of our findings is that the results of AFXa monitoring within 24 h may not be prognostic for patients with CVT, as there was no association with outcomes and most patients were within the therapeutic range within 24 h without requiring dose adjustment.

To our knowledge, there is only one other published study examining AFXa monitoring in the setting of CVT. Saini et al. retrospectively compared aPTT and AFXa assays in 95 children with CVT and reported that peak AFXa and peak aPTT values were not predictive of clinical outcomes, including major bleeding events, which were reported in 5.3% of cases (14). The major bleeding event rate in our adult population (5.9%) was similar.

UFH comprises a diverse blend of glycosaminoglycans that interact with antithrombin (AT) through a pentasaccharide, promoting the deactivation of thrombin and various clotting components. In this context, AT is not directly competing with factor Xa in the assay. Instead, the role of AT is to enhance the inhibitory effect of heparin on factor Xa, resulting in lower levels of unbound factor Xa, which can then be measured by the assay (5). AT enhances the action of heparin in inhibiting factor Xa in the anti-Xa assay by forming a ternary complex with heparin and factor Xa. This assay is used to indirectly measure the residual factor Xa activity in the presence of heparin, providing valuable information for monitoring and adjustment of anticoagulant therapy.

Renal impairment is an important confounding factor because the blood concentration and clearance rate of UFH depend on renal function (7). No patients in our study had renal failure. One patient with a mild AKI (GFR of 57) had a supratherapeutic AFXa while on UFH, and the infusion was stopped as indicated in the guidelines; this patient was transitioned to an alternative anticoagulant. For the purposes of this study, we focused on AFXa levels in patients receiving UFH. Although the issue falls outside the scope of this study, our current clinical practice is to avoid using LMWH in patients with a GFR of less than 30.

In the setting of traumatic injury, findings are inconsistent on whether AFXa monitoring is associated with outcomes. When compared to historical controls, a benefit from AFXa-guided dosing of LMWH has been observed (19, 20). Singer et al. studied 131 trauma patients admitted to the ICU and reported that 35% initially achieved prophylactic levels based on AFXa assay, 25% required a dose adjustment, and 39% did not reach therapeutic levels, but there was a reduction in VTE rates with AFXa-guided dosing to 7.1%, down from 20.5% in historical controls (20). Ko et al. reported lower rates of symptomatic VTE with AFXa dose adjustment of LMWH from 7.6% in historical controls to 1.1% in a study of 205 trauma patients. In contrast, Karcutskie et al. retrospectively studied 792 trauma patients and found no difference in outcomes, including VTE rates with AFXa-guided dosing of thromboprophylaxis with LMWH vs. fixed dosing (6.8% vs. 6.0%). Moreover, 48% of patients never reached prophylactic levels even with TDM (21). In our study, 33% of patients were not within the target therapeutic range within 24 h, and 18% of patients never reached target AFXa levels. While studies in trauma patients are not directly applicable to our population, they shed some light on whether outcomes are improved by achieving higher rates of therapeutic or prophylactic anticoagulation based on AFXa dose adjustments.

Our study does have important limitations to consider. Primarily, these results should be interpreted within the scope of a pilot study because of the small sample size and the fact that all patients were treated at a single center. We were unable to adjust for covariates, and residual confounding was not assessed. A larger, better-powered study would certainly need to employ a multicenter, multiyear design. Second, we did not collect data on resolution of symptoms or modified Rankin scale at discharge. However, discharge disposition was examined, which is an important indicator of clinical outcomes. Third, there was a high transfer rate of 69%, and we acknowledge the related limitations, including not being able to evaluate heparin treatment prior to arrival at our institution. There were seven patients who were therapeutic before starting UFH, six of whom were transfers. Moreover, UFH adjustments may vary by institution, and given the high rate of transfer, variability may have been introduced prior to definitive treatment at our comprehensive stroke center. Fourth, timing (hours) to reach the therapeutic range was calculated as the time from arrival to the first AFXa assay falling within the therapeutic range, rather than from the time when the first dose of UFH was administered, partly because it was not known whether heparin treatment was received at the transferring facility. Fifth, 15 patients were transitioned from UFH to LMWH within 24 h of arrival. A comparison between patients who received UFH, LMWH, or both anticoagulants is provided in Supplementary Table S1. There were no clinical or demographic differences among patients based on the choice of anticoagulant, and there was no difference in outcomes for patients receiving LMWH, UFH, or both. Finally, patients were presumed to have undergone UFH dose adjustments based on AFXa values.

## 5 Conclusion

In this novel pilot study of adults with CVT, there was no association between clinical outcomes and reaching therapeutic levels on UFH, as determined by monitoring of AFXa levels. There was also no association between the time to reach therapeutic AFXa levels and clinical outcomes. Most CVT patients achieved target therapeutic AFXa levels within 24 h of their arrival, and most patients had favorable outcomes. Our findings demonstrate the practicability of monitoring UFH based on AFXa assay in this population of patients with CVT, but reaching target AFXa levels within 24 h of arrival may not necessarily be prognostic.

## Data availability statement

The original contributions presented in the study are included in the article/Supplementary material, further inquiries can be directed to the corresponding author.

## Ethics statement

The studies involving humans were approved by HealthONE institutional review board #779117. The studies were conducted in accordance with the local legislation and institutional requirements. The Ethics Committee/Institutional Review Board waived the requirement of written informed consent for participation from the participants or the participants’ legal guardians/next of kin because this was a minimal-risk retrospective observational study.

## Author contributions

YP: Data curation, Writing – original draft. KS: Formal analysis, Methodology, Software, Writing – original draft. CB: Writing – review & editing. XT: Data curation, Writing – review & editing. DB-O: Writing – review & editing, Project administration, Supervision. RB: Conceptualization, Writing – review & editing.

## References

[1] Field TS Hill MD. Cerebral venous thrombosis. Stroke. (2019) 50:1598–604. doi: 10.1161/STROKEAHA.119.025334, PMID: 31092159

[2] Silvis SM de Sousa DA Ferro JM Coutinho JM. Cerebral venous thrombosis. Nat Rev Neurol. (2017) 13:555–65. doi: 10.1038/nrneurol.2017.104 28820187

[3] Fam D Saposnik G. Critical care management of cerebral venous thrombosis. Curr Opin Crit Care. (2016) 22:113–9. doi: 10.1097/MCC.0000000000000278, PMID: 26645556

[4] Saposnik G Barinagarrementeria F Brown RD Jr Bushnell CD Cucchiara B Cushman M. Diagnosis and management of cerebral venous thrombosis: a statement for healthcare professionals from the American Heart Association/American Stroke Association. Stroke. (2011) 42:1158–92. doi: 10.1161/STR.0b013e31820a8364 21293023

[5] Hirsh J Raschke R. Heparin and low-molecular-weight heparin: the seventh ACCP conference on antithrombotic and thrombolytic therapy. Chest. (2004) 126:188S–203S. doi: 10.1378/chest.126.3_suppl.188S 15383472

[6] Hutt Centeno E Militello M Gomes MP. Anti-Xa assays: what is their role today in antithrombotic therapy? Cleve Clin J Med. (2019) 86:417–25. doi: 10.3949/ccjm.86a.18029, PMID: 31204981

[7] Nutescu EA Burnett A Fanikos J Spinler S Wittkowsky A. Pharmacology of anticoagulants used in the treatment of venous thromboembolism. J Thromb Thrombolysis. (2016) 41:15–31. doi: 10.1007/s11239-015-1314-3, PMID: 26780737 PMC4715843

[8] Juergens CP Semsarian C Keech AC Beller EM Harris PJ. Hemorrhagic complications of intravenous heparin use. Am J Cardiol. (1997) 80:150–4. doi: 10.1016/S0002-9149(97)00309-3 9230150

[9] Hull RD Raskob GE Brant RF Pineo GF Valentine KA. Relation between the time to achieve the lower limit of the APTT therapeutic range and recurrent venous thromboembolism during heparin treatment for deep vein thrombosis. Arch Intern Med. (1997) 157:2562–8. doi: 10.1001/archinte.1997.00440430038005, PMID: 9531224

[10] Kindelin NM Anthes AM Providence SM Zhao X Aspinall SL. Effectiveness of a calculation-free weight-based unfractionated heparin nomogram with anti-Xa level monitoring compared with activated partial thromboplastin time. Ann Pharmacother. (2021) 55:575–83. doi: 10.1177/1060028020961503, PMID: 32964730

[11] Levine MN Hirsh J Gent M Turpie AG Cruickshank M Weitz J. A randomized trial comparing activated thromboplastin time with heparin assay in patients with acute venous thromboembolism requiring large daily doses of heparin. Arch Intern Med. (1994) 154:49–56. doi: 10.1001/archinte.1994.00420010073009, PMID: 8267489

[12] Guervil DJ Rosenberg AF Winterstein AG Harris NS Johns TE Zumberg MS. Activated partial thromboplastin time versus antifactor Xa heparin assay in monitoring unfractionated heparin by continuous intravenous infusion. Ann Pharmacother. (2011) 45:861–8. doi: 10.1345/aph.1Q161, PMID: 21712506

[13] Kitchen S Jennings I Woods TA Preston FE. Wide variability in the sensitivity of APTT reagents for monitoring of heparin dosage. J Clin Pathol. (1996) 49:10–4. doi: 10.1136/jcp.49.1.10, PMID: 8666677 PMC1023149

[14] Saini S Folta AN Harsh KL Stanek JR Dunn AL O’Brien SH. Anti-factor Xa-based monitoring of unfractionated heparin: clinical outcomes in a pediatric cohort. J Pediatr. (2019) 209:212–219.e1. doi: 10.1016/j.jpeds.2019.02.015, PMID: 30961988

[15] Bates SM Weitz JI. Coagulation assays. Circulation. (2005) 112:e53–60. doi: 10.1161/CIRCULATIONAHA.104.478222, PMID: 16043649

[16] Siemens Healthineers. (2023). INNOVANCE Anti-Xa assay. Available at: https://www.siemens-healthineers.com/hemostasis/innovance-assays/innovance-anti-xa

[17] Lee EC Whitehead AL Jacques RM Julious SA. The statistical interpretation of pilot trials: should significance thresholds be reconsidered? BMC Med Res Methodol. (2014) 14:41. doi: 10.1186/1471-2288-14-41, PMID: 24650044 PMC3994566

[18] Serdar CC Cihan M Yucel D Serdar MA. Sample size, power and effect size revisited: simplified and practical approaches in pre-clinical, clinical and laboratory studies. Biochem Med (Zagreb). (2021) 31:010502. doi: 10.11613/BM.2021.010502, PMID: 33380887 PMC7745163

[19] Ko A Harada MY Barmparas G Chung K Mason R Yim DA. Association between enoxaparin dosage adjusted by anti-factor Xa trough level and clinically evident venous thromboembolism after trauma. JAMA Surg. (2016) 151:1006–13. doi: 10.1001/jamasurg.2016.1662, PMID: 27383732

[20] Singer GA Riggi G Karcutskie CA Vaghaiwalla TM Lieberman HM Ginzburg E. Anti-Xa-guided enoxaparin thromboprophylaxis reduces rate of deep venous thromboembolism in high-risk trauma patients. J Trauma Acute Care Surg. (2016) 81:1101–8. doi: 10.1097/TA.0000000000001193, PMID: 27488490

[21] Karcutskie CA Dharmaraja A Patel J Eidelson SA Padiadpu AB Martin AG. Association of Anti-Factor Xa-Guided Dosing of enoxaparin with venous thromboembolism after trauma. JAMA Surg. (2018) 153:144–9. doi: 10.1001/jamasurg.2017.3787, PMID: 29071333 PMC5838588

