# Supplementary material for: Pilot study examining anti-factor Xa levels for heparin monitoring and outcomes in patients with cerebral venous thrombosis
Source: Front Med (Lausanne). 2024 Jan 26;11:1317246. doi: 10.3389/fmed.2024.1317246 (PMC10858448; doi:10.3389/fmed.2024.1317246)
Supplement: Supplementary file 1 [file Table_1.DOCX]

| **Supplementary Table 1.** Association between anticoagulant therapy and outcomes | | | | |
| --- | --- | --- | --- | --- |
| Outcome, n (%) | UFH only | UFH 1^st^, LMWH 2^nd^ | LMWH only | P value |
| Number of patients | 7 | 38 | 52 |  |
| Age, median (IQR) | 40 (33-74) | 43 (34-57) | 46.5 (27-59) | 0.94 |
| Male sex | 2 (28.6) | 19 (50.0) | 19 (36.5) | 0.34 |
| Transfer status, yes | 5 (71.4) | 26 (68.4) | 40 (76.9) | 0.66 |
| Event to arrival, days | 5 (1-10) | 1 (0-3) | 2 (1-3) | 0.32 |
| Endovascular therapy | 3 (42.9) | 19 (50.0) | 27 (51.9) | 0.90 |
| Disposition |  |  |  | 0.31 |
| Home | 4 (57.1) | 31 (81.6) | 41 (78.9) |  |
| LTAC/Rehab | 2 (28.6) | 7 (18.4) | 8 (15.4) |  |
| Died | 1 (14.3) | 0 (0) | 3 (5.8) |  |
| Median LOS (IQR) | 6 (3-13) | 5 (4-8) | 4 (3-6) | 0.49 |

UFH, unfractionated heparin; LMWH, low molecular weight heparin; LTAC, long term acute care facility; LOS, length of stay; IQR, interquartile range
